# Experimental verification of a broadband asymmetric transmission metamaterial in the terahertz region

**DOI:** 10.1039/c9ra10861k

**Published:** 2020-02-10

**Authors:** Xiang Tao, Limei Qi, Jun Yang, Fanyi Liu

**Affiliations:** School of Electronic Engineering, Beijing University of Posts and Telecommunications Beijing 100876 China qilimei1204@163.com; School of Information and Communication Engineering, Beijing University of Posts and Telecommunications Beijing 100876 China

## Abstract

In this work, a broadband terahertz asymmetric transmission metamaterial is experimentally demonstrated for a linearly polarized wave. The measured transmission coefficient *T*_*yx*_ is larger than 0.6 from 0.55 to 0.82 THz, and reaches a peak value of 0.714 at 0.62 THz, while the transmission coefficient *T*_*xy*_ is lower than 0.2 from 0.4 to 0.9 THz. The calculated asymmetric transmission parameter of the measurement ranges from 0.53 to 0.84 THz for magnitudes over 0.4. The peak value reached 0.65 at the frequency of 0.78 THz. The physical mechanism of the polarization conversion was also analyzed from the distributions of the surface currents and electric fields.

## Abstract 2

In this work, a broadband terahertz asymmetric transmission metamaterial is experimentally demonstrated for a linearly polarized wave.

## Graphical abstract

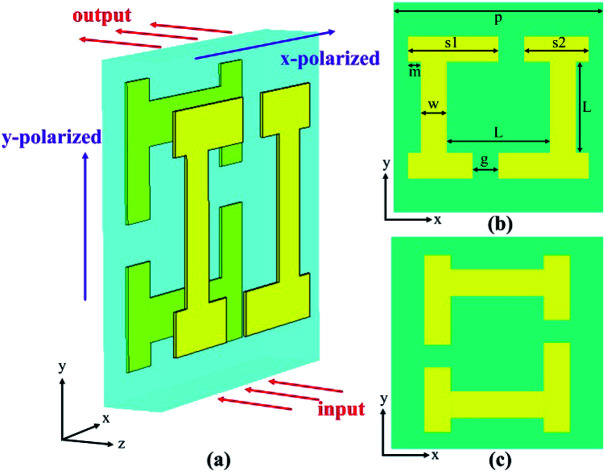

## 1. Introduction

Metamaterials possess novel electromagnetic properties and can be periodically structured with unit cells. This kind of new material has many potential applications, such as in negative refraction index materials, perfect lenses, and invisibility cloaks.^1–3^ Since Pendry *et al.* reported negative refraction by utilizing chirality in 2004,^4^ chiral metamaterials have attracted the attention of many researchers. Many new properties have been found in the chiral metamaterials, such as asymmetric transmission (AT), circular dichroism and optical activity. Asymmetric transmission, defined as the difference between the transmittance in two opposite propagation directions, has been largely reported on planar metasurfaces in microwave,^5–12^ THz,^13–20^ infrared^21^ and optical^22–28^ regimes. It was found that a chiral metamaterial with symmetry breaking can exhibit an AT effect for linear and circular polarizations.^10,27–32^ For the experimental observations of AT, several metamaterials with AT effects are reported. In the microwave band, Mutlu *et al.*^7^ demonstrated a narrow asymmetric transmission of linear polarization at 7.1 GHz with *T*_*yx*_ = 0.171 and *T*_*xy*_ = 0.974. Huang *et al.*^8^ presented a measurement of the chiral metamaterial AT structure with a *T*_*yx*_ value that reached a maximum of approximately 0.8 at 10.24 GHz and the *T*_*xy*_ was a small value of approximately 0.02. Cheng *et al.*^9^ reported a strong AT effect for the linear polarization. The cross-polarization transmission *T*_*xy*_ achieves a maximum of 0.74 in the experiment and 0.77 in the simulation at an approximate resonance frequency of 9.65 GHz, although *T*_*xy*_ is very small and remains below 0.1 over the entire frequency range. Shi *et al.*^10^ revealed a narrow dual-band AT transmission at about 10.79 and 14.57 GHz. It was demonstrated that the *T*_*xy*_ reached a maximum of 0.95 at around 10.79 GHz and the *T*_*yx*_ was below 0.15. Meanwhile, an obvious resonant peak for *T*_*yx*_ was larger than 0.93 at around 14.57 GHz with a *T*_*xy*_ value that was below 0.15. In addition, Wei *et al.*^30^ demonstrated a broadband multilayer stacked AT metamaterial, and the measured *T*_*yx*_ was greater than 0.96 within a frequency range of 9.8–12.5 GHz. In the infrared band, a monolayer all-dielectric metasurface was proposed to realize broad circular asymmetric transmission with an AT parameter of 0.69 at 9.6 μm.^21^ In the optical band, Menzel *et al.*^23^ reported the first experimental observation and theoretical analysis of AT in a 3D low-symmetry metamaterial. The difference between the *T*_*xy*_ and *T*_*yx*_ achieved values of up to 25% in the linear base. Wang *et al.*^31^ fabricated an AT metamaterial in the near-infrared region. It was found that only the forward direction was allowed for the *x* polarization at around 1350 nm, and only the backwards direction was allowed for the *y* polarization. Pfeiffer *et al.*^32^ reported a metasurface with an AT of circularly polarized light at a wavelength of 1.5 μm. The experimental transmittance and extinction ratio were 50% and 20 : 1, respectively.

In the terahertz region, Singh *et al.*^13^ presented experimental and numerical evidence of the AT of a circularly polarized terahertz wave through a planar chiral metamaterial for the first time. Then, Kenney *et al.*^16^ fabricated a herringbone metasurface to realize a broadband asymmetry between the orthogonal circular polarizations with a cross-polarization transmittance of 0.62. Recently, Liu *et al.*^17^ demonstrated a temperature-controlled AT of linearly polarized THz waves by exploiting the insulator-to-metal phase transition of VO_2_. In this work, a two-dimensional chiral structure with broken symmetry was experimentally demonstrated to realize a broadband AT effect for linearly polarized waves in the terahertz band. It consisted of two metallic split rectangular annulus on two sides of a dielectric layer. The measured transmission coefficient *T*_*yx*_ is larger than 0.6 from 0.55 to 0.82 THz, and reached a peak value of 0.714 at 0.62 THz, although the transmission coefficient *T*_*xy*_ was lower than 0.2 from 0.4 to 0.9 THz. The physical mechanism of the polarization conversion was also analyzed using the electric field distributions.

## 2. Designed structure and simulations

**Fig. 1:**
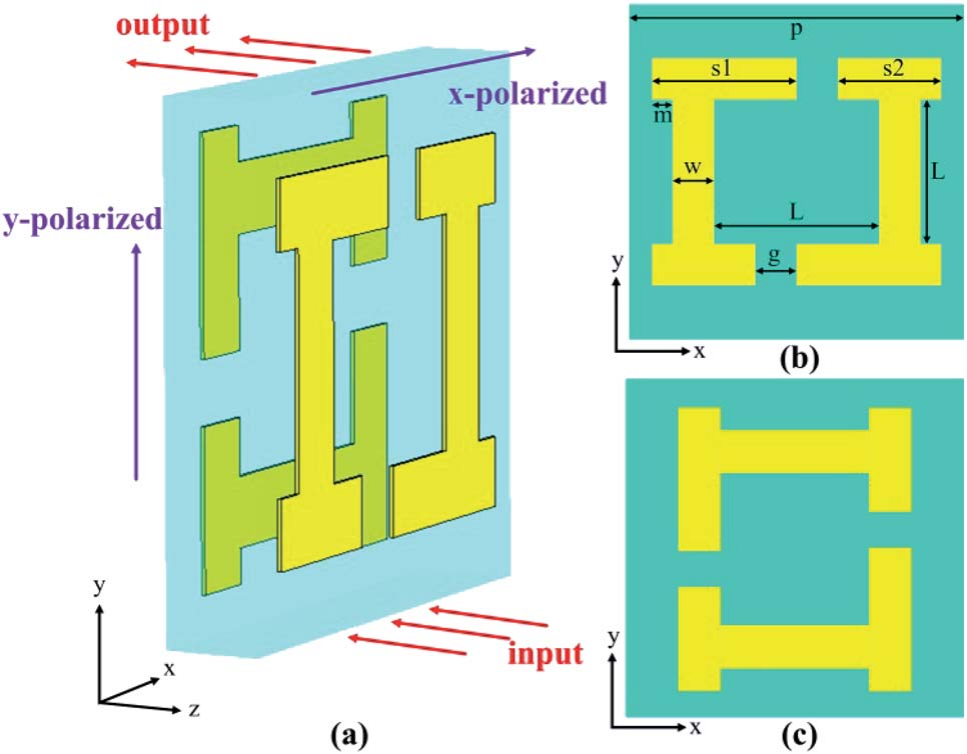
Geometry of the designed structure. (a) The perspective view of the unit cell, (b) the top metal structure and (c) bottom metal structure from the top view of the structure.

Fig. 1a shows the unit cell of the designed chiral metamaterial, which is composed of double I-shape metallic structures on two sides of a dielectric substrate. The metallic patterns on both sides of the dielectric layer are identical but twisted. As is shown in Fig. 1b and c, the bottom metal structure is formed by rotating the top metal structure with a clockwise angle of 90° along the *z*-axis and then this is mirrored along the *y*-axis. The dielectric substrate is made of polyimide with a relative permittivity of *ε*_r_ = 3.4 and a loss tangent of tg *δ* = 0.008. The substrate thickness was *d* = 25 μm. Other parameters of the unit cell are as follows: *p* = 268 μm, *L* = 119 μm, *w* = 35.5 μm, *g* = 33 μm, *s*_1_ = 110.5 μm, *s*_2_ = 82.5 μm, and *m* = 18 μm. The thickness of the gold is *t* = 0.1 μm. Commercial software (CST Microwave Studio) was used for the simulation. An open boundary condition was employed along the *z* axis and unit cell boundary conditions were employed along the *x* and *y* axes. Adaptive tetrahedral mesh refinement was used to ensure the accuracy of the simulation. The electromagnetic wave can be divided into the *x*-polarized wave in which the electric field is parallel to the *x*-axis and the *y*-polarized wave in which the electric field is parallel to the *y*-axis (as shown in Fig. 1a).

Eqn (1) is used to describe the transmitted electric field of a linearly polarized wave. The complex amplitudes of the incident and the transmitted waves are given in eqn (2).^8^ In which *T*_*xx*_ represents the transmission coefficient of the transmitted waves polarized on the *x* direction when the incident wave is *x*-polarized, while *T*_*yx*_ represents the transmission coefficient of the transmitted waves polarized in the *y* direction when the incident wave is *x*-polarized, *T*_*yy*_ and *T*_*xy*_ are defined in the same way.

1 *E*_t_(*r*, *t*) = (*T*_*x*_, *T*_*y*_)^*T*^e^*j*(*kz*−*wt*)^

2
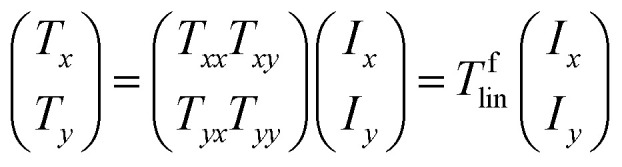


**Fig. 2:**
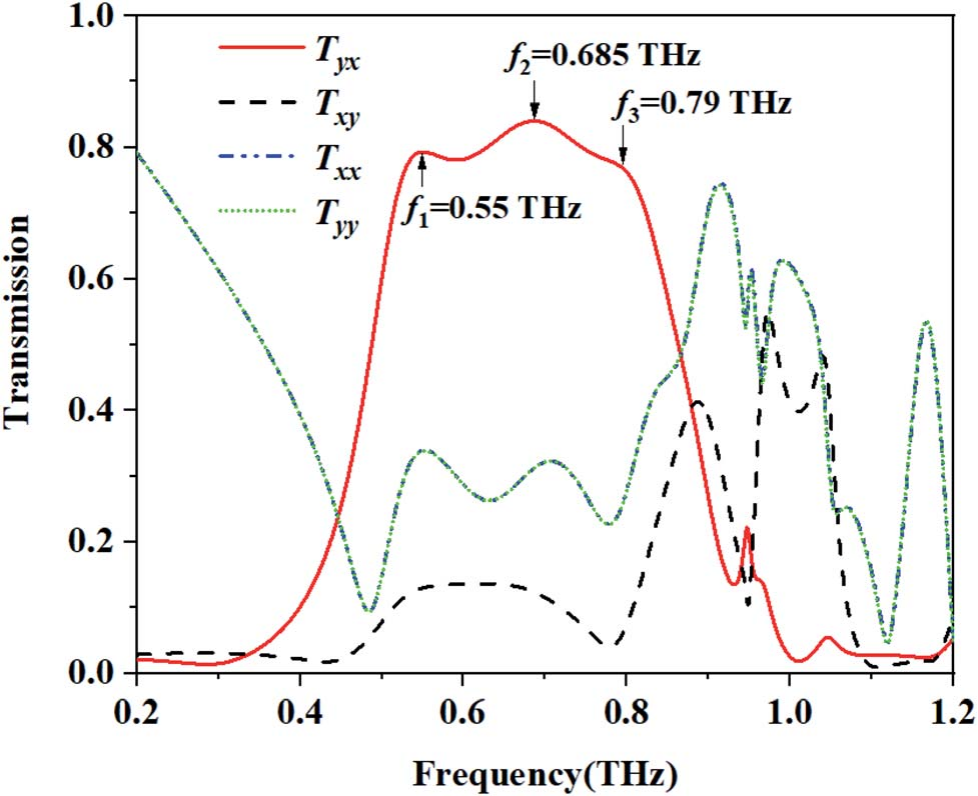
The simulated transmission coefficients.

Fig. 2 shows the simulated transmission coefficients of a linearly polarized incident wave. It is obvious that the cross-polarized transmission coefficient *T*_*xy*_ is different from *T*_*yx*_, while the co-polarized transmission coefficients *T*_*xx*_ and *T*_*yy*_ always remain equal across the frequency ranges. In addition to this, we also found that the transmission coefficient *T*_*yx*_ can reach three peak values of 0.795, 0.84 and 0.773 at *f*_1_ = 0.55 THz, *f*_2_ = 0.685 THz and *f*_3_ = 0.79 THz, respectively. The magnitude of *T*_*yx*_ is larger than 0.707 across a wide range from 0.51 to 0.84 THz with a relative bandwidth of about 50%, while the magnitude of *T*_*xy*_ is lower than 0.2 from 0.51 to 0.84 THz. Owing to the difference between *T*_*xy*_ and *T*_*yx*_, a broadband asymmetric transmission is achieved.

The asymmetric transmission for a certain polarization state can be defined as the difference between the transmittance in two opposite propagation directions. For the linearly polarized wave, the AT parameter can be described as:^33^3
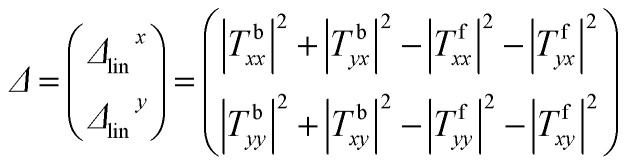


**Fig. 3:**
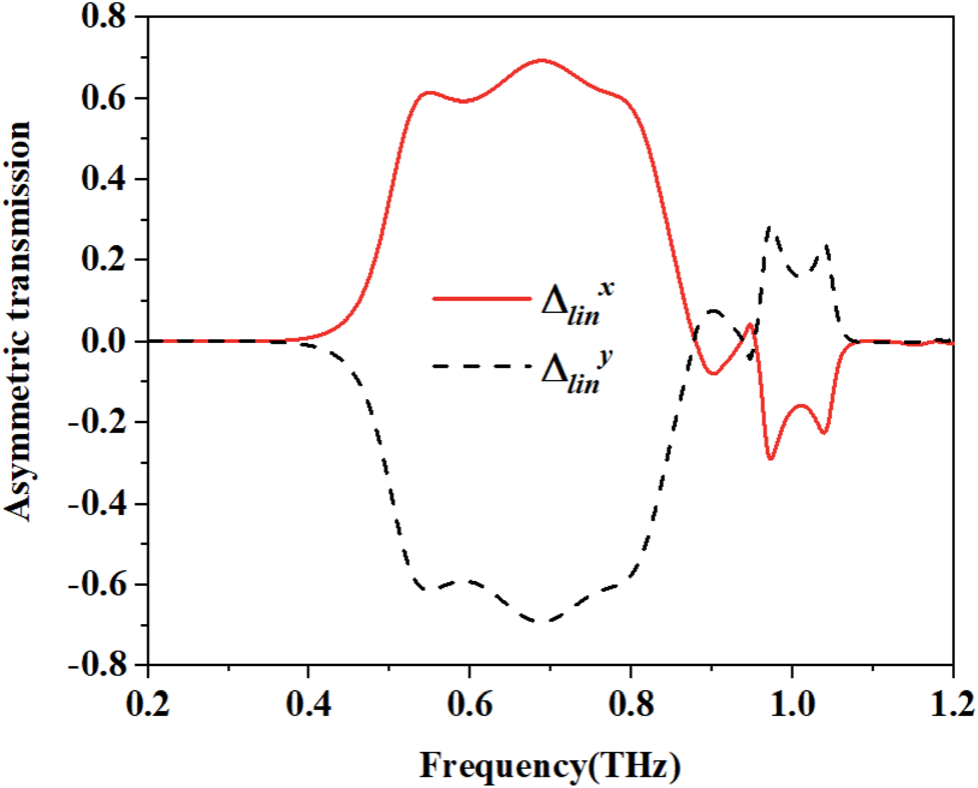
Calculated asymmetric transmission (AT) parameters.

In which the superscript f and the superscript b indicate the wave propagation in the forward (+*z*) and backward (−*z*) directions, respectively. For the structure with reciprocity properties, the two incident waves from the forward and backwards directions possess the same transmission coefficients with respect to their polarization direction. In which *T*^b^_*xx*_ = *T*^f^_*xx*_, *T*^b^_*yx*_ = *T*^f^_*xy*_, *T*^b^_*yy*_ = *T*^f^_*yy*_ and *T*^b^_*xy*_ = *T*^f^_*yx*_. Then, the two curves of *Δ*_lin_^*x*^ and *Δ*_lin_^*y*^ are completely identical and opposite to each other. The calculated AT parameters *Δ*_lin_^*x*^ and *Δ*_lin_^*y*^ are shown in Fig. 3. The AT parameters range from 0.533 to 0.794 THz for the high magnitude over 0.59. The peak value reaches 0.693 at a frequency of 0.687 THz. These results verify that our proposed structure can achieve a broadband asymmetric transmission with a linearly polarized wave.

4 *T*_*x*_ = |*T*_*xx*_|^2^ + |*T*_*yx*_|^2^

5 *T*_*y*_ = |*T*_*yy*_|^2^ + |*T*_*xy*_|^2^

6 PCR_*x*_ = |*T*_*yx*_|^2^/(|*T*_*xx*_|^2^ + |*T*_*yx*_|^2^)

7 PCR_*y*_ = |*T*_*xy*_|^2^/(|*T*_*yy*_|^2^ + |*T*_*xy*_|^2^)

**Fig. 4:**
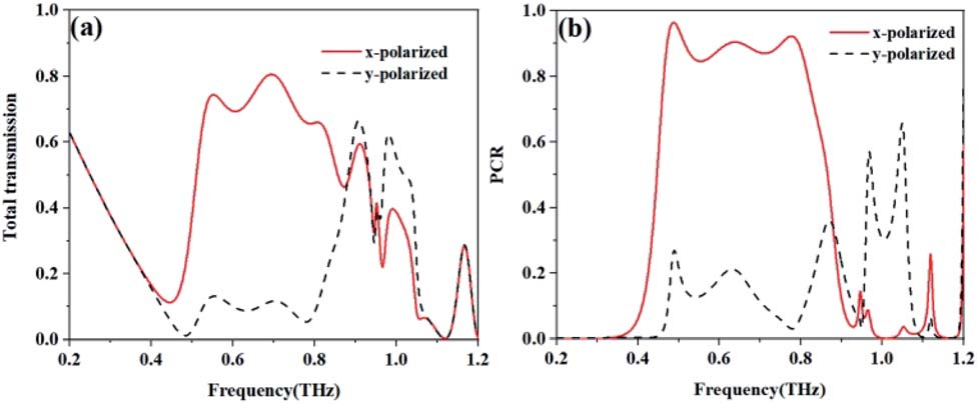
The calculated (a) total transmission and (b) PCR.

Fig. 4a shows the total transmissions (based on eqn (4) and (5)) of the *x*-polarized and *y*-polarized wave propagation along the input (−*z*) direction. The total transmission of the *x*-polarized waves range from 0.53 to 0.752 THz with the magnitude value above 0.7 and reached maximum values of 0.806 at 0.693 THz, while the total transmission of the *y*-polarized wave was lower than 0.15 from 0.52 to 0.825 THz. From Fig. 4a, it is obvious that the *x*-polarized incident wave can transmit well through the proposed structure, while most of the *y*-polarized incident waves are forbidden along the input (−*z*) direction. If the incident wave propagates along the +*z* direction, the result is completely opposite (not shown).

As shown in Fig. 4b, the polarization conversion ratios (PCR) of the *x*-polarized and the *y*-polarized waves are calculated based on eqn (6) and (7). The polarization conversion ratio of the *x*-polarized wave is higher than 0.8 from 0.463 to 0.817 THz and reaches a peak of 0.964 at 0.487 THz. Meanwhile, the polarization conversion ratio of the *y*-polarized wave can reach a minimum value of 0.03 and a maximum value of 0.36 at 0.778 and 0.871 THz, respectively. These results mean the structure can realize a high polarization conversion efficiency for the *x*-polarized wave over a broadband frequency range when the incident wave propagates along the −*z* direction.

**Fig. 5:**
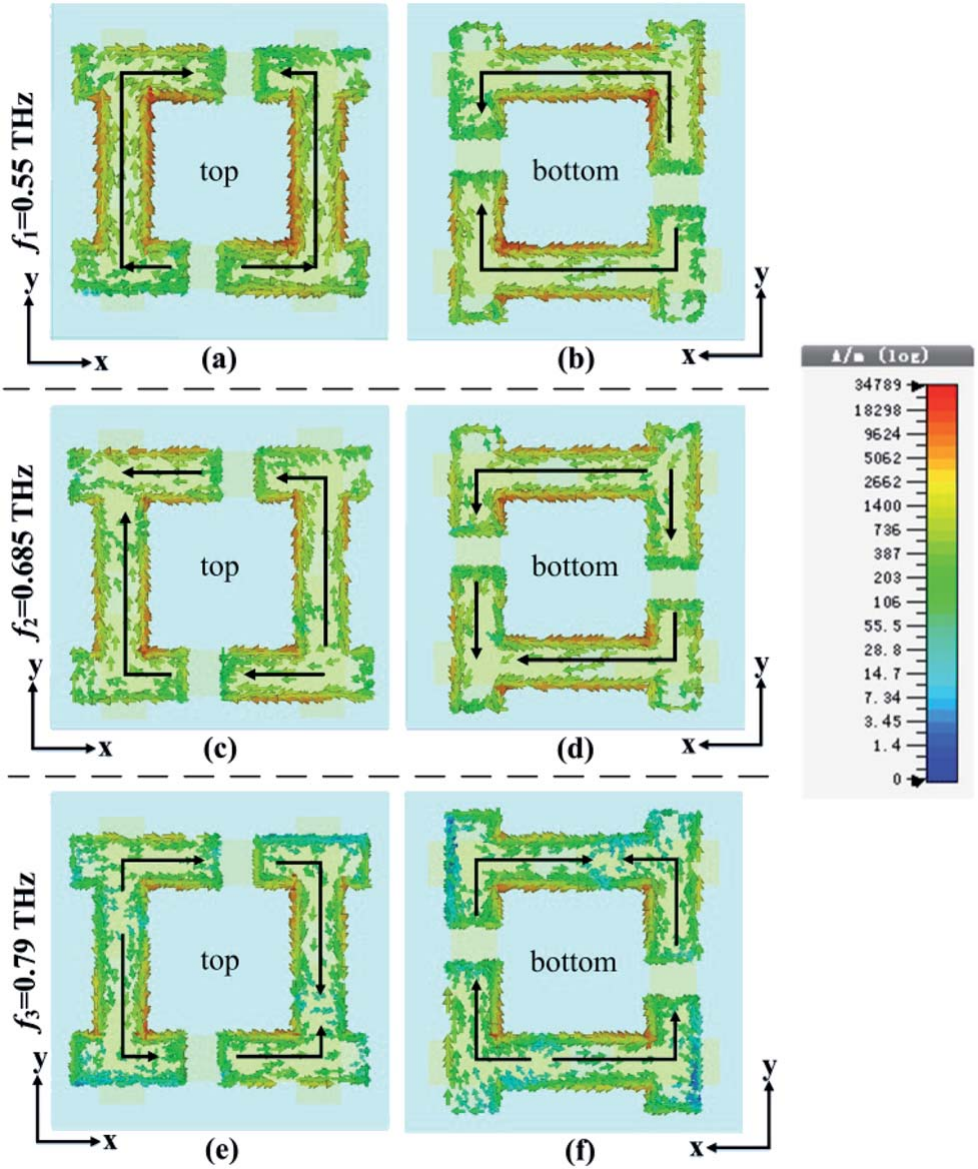
The surface current of the *x*-polarized wave on the top and bottom metals at *f*_1_ = 0.55 THz, *f*_2_ = 0.685 THz and *f*_3_ = 0.79 THz, respectively. (a), (c) and (e) show the top surface currents. (b), (d) and (f) show the bottom surface currents.

The surface current of the proposed structure was simulated to better explain the AT effect. Fig. 5 shows the surface current distribution on the top and bottom metallic layers of the *x*-polarized wave as it propagates along the −*z* direction at *f*_1_ = 0.55 THz, *f*_2_ = 0.685 THz and *f*_3_ = 0.79 THz, the black solid arrows represent the direction of the current. From Fig. 5a and b, it can be seen that the direction of the current is the same for each I-shape metallic structure at *f*_1_ = 0.55 THz, which means the current direction mode oscillates in phase at this resonate frequency. From Fig. 5c and d, it can be seen that there are two different current direction modes that exist on each I-shape metallic structure at *f*_2_ = 0.685 THz, and the two current direction modes oscillate out of phase at the resonate frequency. From Fig. 5e and f, it can be seen that the current direction mode oscillates out of phase at *f*_3_ = 0.79 THz. We believe that the different transmissions at *f*_1_ = 0.55 THz, *f*_2_ = 0.685 THz and *f*_3_ = 0.79 THz are all caused by these in phase and out of phase current direction modes.^34,35^

**Fig. 6:**
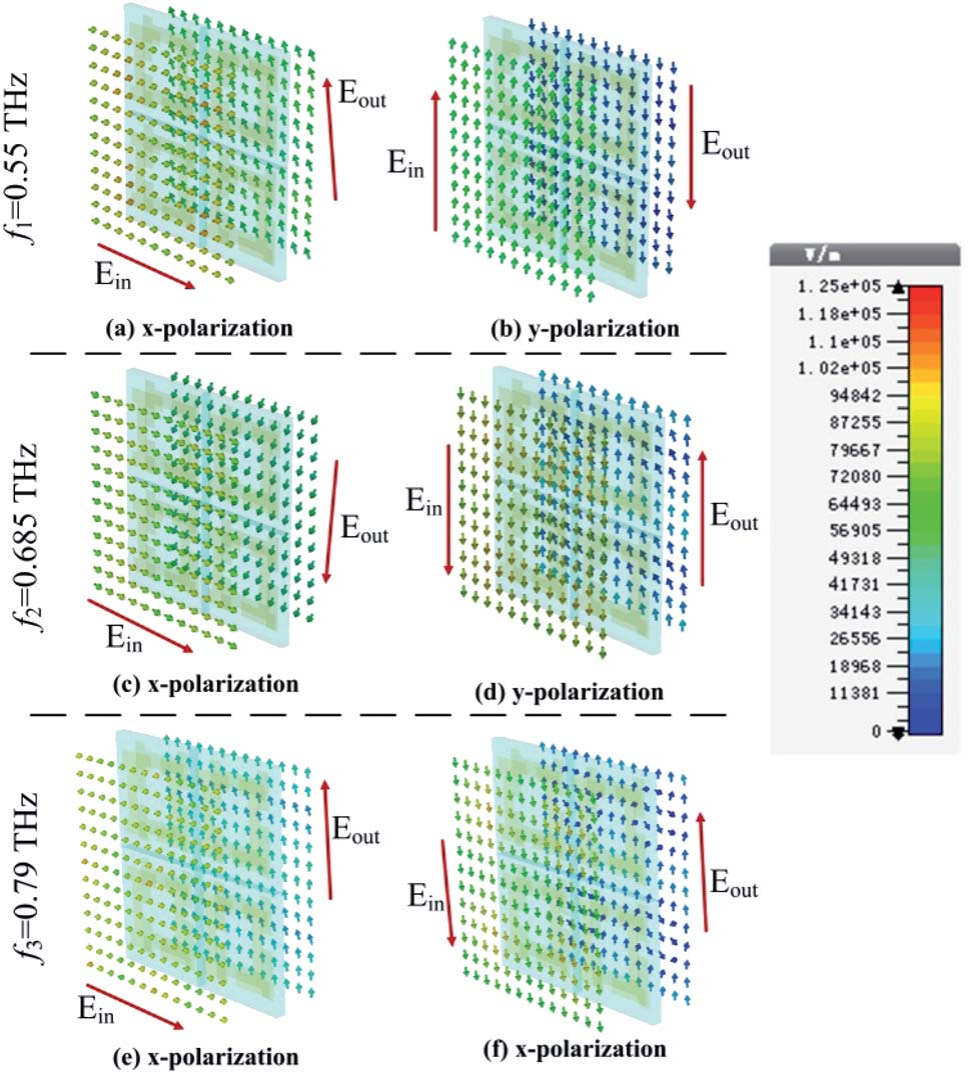
Variation in the electric field directions when the *x*-polarized and *y*-polarized waves propagate in the −*z* direction at *f*_1_ = 0.55, *f*_2_ = 0.685 and *f*_3_ = 0.79 THz, respectively.

To investigate the mechanism for the AT that is associated with the chiral metamaterial, Fig. 6 shows the electric field distributions for the *x*-polarized wave passing through the AT structure backwards (−*z*) at *f*_1_ = 0.55 THz, *f*_2_ = 0.685 THz and *f*_3_ = 0.79 THz, respectively. For *f*_1_ = 0.55 THz in Fig. 6a and b, the electric field of the *x*-polarized wave in the input plane rotates by about 90° after it arrives at the output plane, and the electric field of the *y*-polarized wave rotates about 180° in the output plane. As a result, most *x*-polarized waves change into a cross-polarization wave and the *y*-polarized wave shows little cross polarization at *f*_1_ = 0.55 THz, respectively. For the electric field distributions at *f*_2_ = 0.685 THz and *f*_3_ = 0.79 THz, a similar phenomenon can be observed. In addition, in Fig. 6b, d and f, most energies are forbidden when the *y*-polarized wave passes through the proposed AT structure in the backwards (−*z*) direction, which is coincident with the black dashed line shown in Fig. 4a. However, in Fig. 6a, c and e, most electric fields of the *x*-polarized wave are rotated by about 90° with a high magnitude. Therefore, as shown in Fig. 4a, the total transmission of the *x*-polarized wave is 0.745, 0.803 and 0.66 at 0.55, 0.685 and 0.79 THz, respectively, while the total transmission of the *y*-polarized wave is 0.131, 0.112 and 0.062 at 0.55, 0.685 and 0.79 THz, respectively.

## 3. Experimental results and discussions

**Fig. 7:**
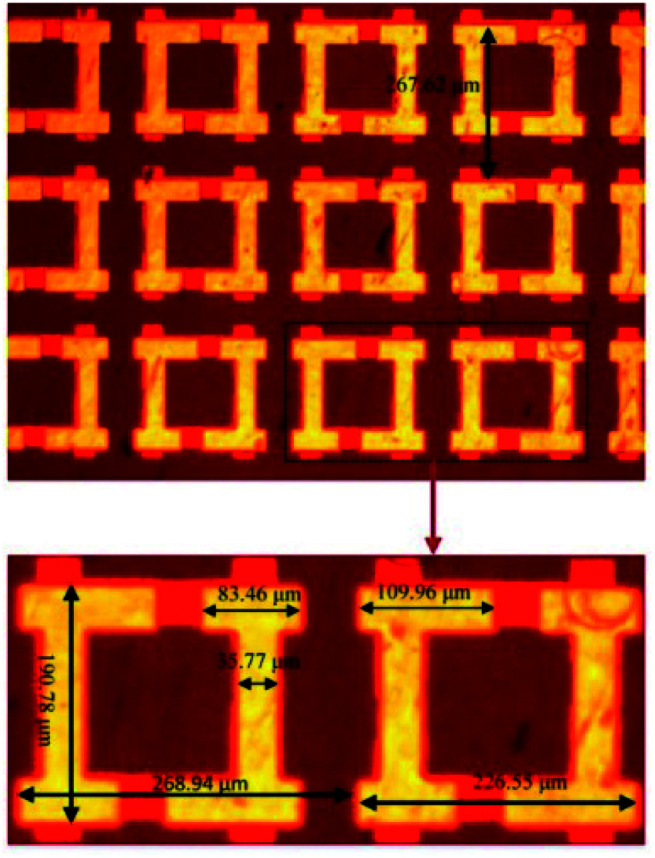
Optical micrograph of the fabricated AT metamaterial.

In order to verify the asymmetric transmission validity of the proposed AT metamaterial. The sample was fabricated using the standard lithography technique and tested using terahertz time-domain-spectroscopy. The sample was made by using two different masks. To keep the front and back metallic patterns in alignment, marks were made on the two masks. Firstly, a 10 nm/100 nm thick Ti/Au film was deposited on one side of the 25 μm polyimide, and a lift off process was used to form the metallic patterns. Then, the same metalized process was used on the other side of the polyimide. The fabricated AT metamaterial had a 40 × 40 square array cell with a period of 250 μm. A good uniformity was achieved across the 10 × 10 mm device area. Fig. 7 shows the optical micrograph of the fabricated AT metamaterial, in which the corresponding diameters are also shown. The dark areas are the dielectric layer areas, and the lighter area is the gold coating.

**Fig. 8:**
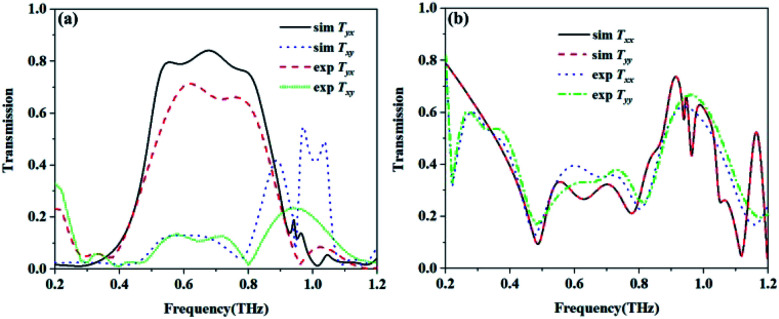
The simulated and measured transmission coefficients. (a) The cross polarization transmission *T*_*yx*_ and *T*_*xy*_, and (b) the co-polarization transmission *T*_*xx*_ and *T*_*yy*_.

**Fig. 9:**
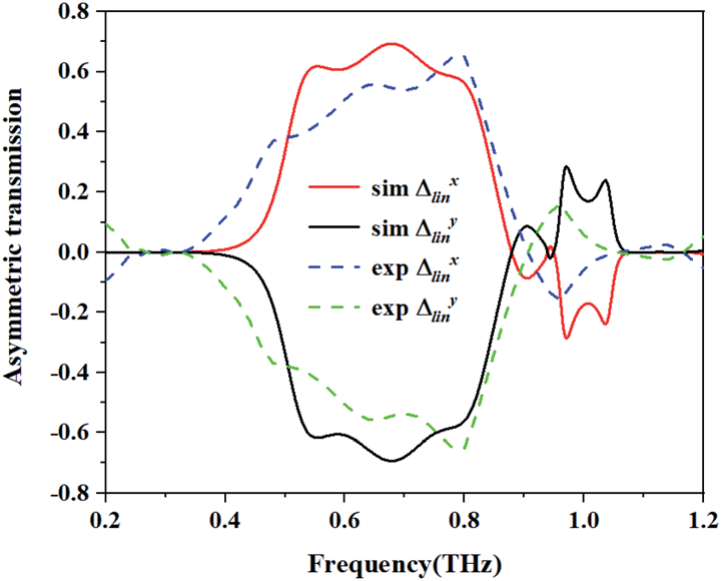
The calculated AT parameters for the measurements and simulation.

Fig. 8 shows the simulated and the measured transmission coefficients of the sample. The simulation parameters were obtained from the measurement of the sample, and were a little different from those used in Fig. 2. Here, *p* = 268.94 μm, *L* = 119.24 μm, *w* = 35.77 μm, *g* = 33.13 μm, *s*_1_ = 109.96 μm, *s*_2_ = 83.46 μm, and *m* = 17.885 μm. From Fig. 8a, we can see that the measured results are essentially consistent with the simulated results. For the measurement, it is obvious that the cross-polarized transmission coefficient *T*_*xy*_ (dashed line) is different from *T*_*yx*_ (short dotted line). The transmission coefficient *T*_*yx*_ is larger than 0.6 in the range from 0.55 to 0.82 THz, and reached a peak value of 0.714 at 0.62 THz, while the transmission coefficient *T*_*xy*_ is lower than 0.2 in the range from 0.4 to 0.9 THz. Fig. 8b shows the co-polarization transmission of the simulated and measured results, it should be noted that the co-polarization transmission *T*_*xx*_ and *T*_*yy*_ of the measured results are almost equal to those of the simulation results. The calculated AT parameters for the measurements and simulations are given in Fig. 9 and are denoted by the solid and the dashed lines, respectively. The two curves of *Δ*_lin_^*x*^ and *Δ*_lin_^*y*^ are identical and opposite to each other. For this measurement, the AT parameter for the measurement ranges from 0.53 to 0.84 THz were used for magnitudes over 0.4. The peak value reached 0.65 at a frequency of 0.78 THz, which is slightly lower than that found using the simulation results.

**Fig. 10:**
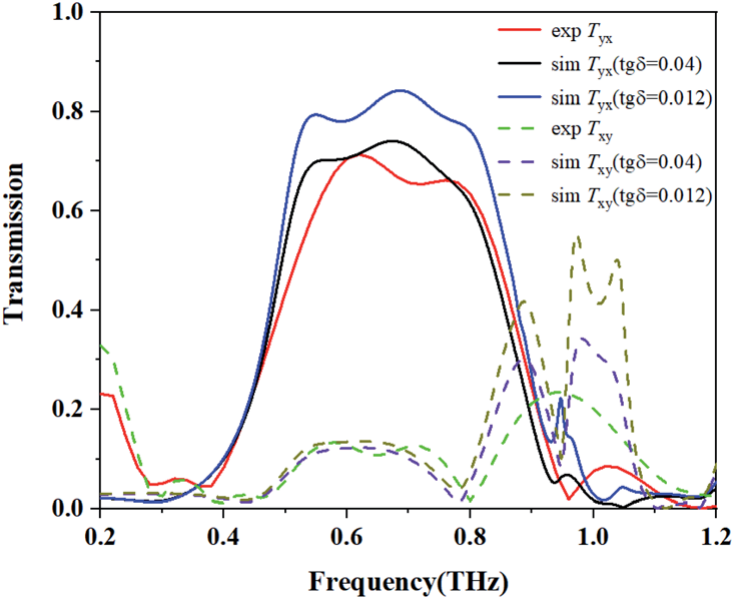
Influence of the tangential loss of polyimide on *T*_*yx*_.

There are several possible causes for the difference between the measured and simulated results. Firstly, the front and back metal structures are aligned in the simulation, while in the sample the front and back metals may be offset. Secondly, owing to the tolerance of size during fabrication, a difference between the measured and simulated results will occur. Thirdly, during the testing process, the sample has to be rotated 90 degrees by hand to measure the result of another polarization state. Some unavoidable position errors may occur, which would result in further errors. For the polyimide, the tangential loss tg *δ* = 0.008 provided by the manufacturer was used in the simulation. However, some deviation in this value may exist. Fig. 10 shows the influence of the tangential loss on the cross-polarization for tg *δ* = 0.012 and 0.04. It can be seen that the magnitude of *T*_*yx*_ decreases with the increasing value of tg *δ*, the simulated result for tg *δ* = 0.04 is closer to the measured result. All of the reasons discussed above may cause differences between the measured and simulated results.

## 4. Conclusions

In this paper, a bi-layered chiral structure consisting of a double I-shape metallic structure on two sides of a dielectric substrate was proposed and experimentally demonstrated, and a high-efficiency and broadband polarization conversion of the linearly polarized wave in the terahertz band was realized. When the linearly polarized waves propagate in the input (−*z*) directions, the simulated transmission coefficient *T*_*yx*_ is more than 0.707 across a wide range from 0.51 to 0.84 THz, and the relative bandwidth is about 50%. When the polarization conversion ratio of the *x*-polarized wave is more than 0.85 from 0.47 to 0.808 THz it reaches a maximum value of 0.964 at 0.488 THz. The measured transmission coefficient *T*_*yx*_ is larger than 0.6 from 0.55 to 0.82 THz, and reaches a peak value of 0.714 at 0.62 THz, while the transmission coefficient *T*_*xy*_ is lower than 0.2 from 0.4 to 0.9 THz. The physical mechanism of the asymmetric transmission and the polarization conversion was also analyzed using the electric field distributions and the surface current distributions.

## Conflicts of interest

There are no conflicts to declare.

## Supplementary Material

## References

[cit1] Smith D. R., Pendry J. B., Wiltshire M. C. K. (2004). Science.

[cit2] Fang N., Lee H., Sun C., Zhang X. (2005). Science.

[cit3] Liu R., Ji C., Mock J. J., Chin J. Y., Cui T. J., Smith D. R. (2009). Science.

[cit4] Pendry J. B. (2004). Science.

[cit5] Mutlu M., Akosman A. E., Serebryannikov A. E., Ozbay E. (2011). Opt. Express.

[cit6] Kang M., Chen J., Cui H. X., Li Y., Wang H. T. (2011). Opt. Express.

[cit7] Mutlu M., Akosman A. E., Serebryannikov A. E., Ozbay E. (2012). Phys. Rev. Lett..

[cit8] Huang C., Feng Y., Zhao J., Wang Z., Jiang T. (2012). Phys. Rev. B: Condens. Matter Mater. Phys..

[cit9] Cheng Y., Nie Y., Wang X., Gong R. (2013). Appl. Phys. A.

[cit10] Shi J. H., Liu X., Yu S., Lv T., Zhu Z., Ma H., Cui T. J. (2013). Appl. Phys. Lett..

[cit11] Cheng Y. Z., He B., Wu C. J., Gong R. Z. (2016). Mater. Sci. Forum.

[cit12] Cheng Y. Z., Zhao J. C., Mao X., Gong R. (2017). Prog. Electromagn. Res..

[cit13] Singh R., Plum E., Menzel C., Rockstuhl C., Azad A. K., Cheville R. A., Lederer F., Zhang W., Zheludev N. I. (2009). Phys. Rev. B: Condens. Matter Mater. Phys..

[cit14] Liu D., Xiao Z., Ma X., Ma Q., Xu X., Wang Z. (2015). Opt. Commun..

[cit15] Li X. F., Feng R., Ding W. Q. (2018). J. Phys. D: Appl. Phys..

[cit16] Kenney M., Li S., Zhang X., Su X., Kim T. T., Wang D., Sun H. (2016). Adv. Mater..

[cit17] Liu M., Xu Q., Chen X., Plum E., Li H., Zhang X., Zhang W. (2019). Sci. Rep..

[cit18] Cheng Y. Z., Gong R. Z., Wu L. (2017). Plasmonics.

[cit19] Cheng Y. Z., Fan J. P., Luo H., Chen F., Feng N. X., Mao X. S., Gong R. Z. (2019). Opt. Mater. Express.

[cit20] Cheng Y. Z., Luo H., Chen F., Mao X. S., Gong R. Z. (2019). OSA Continuum.

[cit21] Zhang F., Pu M., Li X., Gao P., Ma X., Luo J., Luo X. (2017). Adv. Funct. Mater..

[cit22] Fedotov V. A., Schwanecke A. S., Zheludev N. I., Khardikov V. V., Prosvirnin S. L. (2007). Nano Lett..

[cit23] Menzel C., Helgert C., Rockstuhl C., Kley E. B., Tünnermann A., Pertsch T., Lederer F. (2010). Phys. Rev. Lett..

[cit24] Xu Y., Shi Q., Zhu Z., Shi J. H. (2014). Opt. Express.

[cit25] Peng N., She W. (2014). Opt. Express.

[cit26] Wang Y., Wen X., Qu Y., Wang L., Wan R., Zhang Z. (2016). Opt. Express.

[cit27] Tang D. F., Wang C., Pan W. K., Li M. H., Dong J. F. (2017). Opt. Express.

[cit28] Zhao J., Fu Y., Liu Z., Zhou J. (2017). Opt. Express.

[cit29] Kang M M., Chen J., Cui H. X., Li Y., Wang H. T. (2011). Opt. Express.

[cit30] Wei Z., Cao Y., Fan Y., Yu X., Li H. (2011). Appl. Phys. Lett..

[cit31] Wang Y., Kim I., Jin R. C. (2018). RSC Adv..

[cit32] Pfeiffer C., Zhang C., Ray V. (2014). Phys. Rev. Lett..

[cit33] Mutlu M., Akosman A. E., Serebryannikov A. E., Ozbay E. (2012). Phys. Rev. Lett..

[cit34] Liu N., Giessen H. (2010). Angew. Chem., Int. Ed..

[cit35] Schurig D., Mock J. J., Smith D. R. (2006). Appl. Phys. Lett..

